# The Alleged Employment of Chloroform by Thieves

**Published:** 1850-05

**Authors:** John Snow


*The alleged employment of Chloroform by thieves.* By JOHN SNOW, M. D.—In two recent cases of robbery it has been asserted that chloroform was used to render the victims insensible; and although no real evidence has appeared of such being the fact, yet the statement has gained great publicity through the newspapers, and the sentences on the prisoners have apparently been rendered more severe by the allegation.

It can readily be shown that if thieves and prostitutes were to resort to the use of chloroform in the public streets, in the manner alleged, the attempt would only lead to their instant detection on the spot. The sensation of pungency in the nostrils and throat that is caused by this agent, when its vapour is in sufficient quantity to produce any effect on the sensorium, is so strong and peculiar, that no person can take a single inspiration without being aware that he is inhaling something very unusual. Chloroform, in fact, can never be administered without the consent of the party taking it, unless by main force, which has to be used in the case of children who are not old enough to be reasoned into taking it. If a child be asleep when the process of inhalation is commenced, it nearly always awakes before being made insensible, however gently the vapour may be insinuated. As breathing is perfectly under the control of the will, a person would, on finding such a strange attempt being made on him in the public street, instantly hold his breath, and use all his powers of resistance to repel the assault. And supposing the handkerchief, which is the alleged weapon, were held forcibly over his mouth and nostrils, in spite of his efforts, yet he would be able to struggle as long, whilst holding his own breath, as if another person were trying to prevent his breathing by the method called Burking. When it is recollected that a race of 150 yards can be run in one breath, these struggles, it will be perceived, would last long enough to attract a crowd.

*London Medical Gazette.*

